# Comparison of posterior muscle-preserving selective laminectomy and laminectomy with fusion for treating cervical spondylotic myelopathy: study protocol for a randomized controlled trial

**DOI:** 10.1186/s13063-023-07123-4

**Published:** 2023-02-11

**Authors:** Anna MacDowall, Håkan Löfgren, Erik Edström, Helena Brisby, Catharina Parai, Adrian Elmi-Terander

**Affiliations:** 1 Department of Surgical Sciences, Uppsala University, Entrance 61, 6th floor, 75185 Uppsala, Sweden; 2 Neuro-Orthopedic Center, Jönköping, Jönköping County, and Department of Biomedical and Clinical Sciences, Linköping University, Linköping, Sweden; 3 Department of Neurosurgery, Karolinska University Hospital, Stockholm, Sweden; 4 Department of Clinical Neuroscience, Karolinska Institutet, Stockholm, Sweden; 5 Capio, Spine Center Stockholm, Upplands-Väsby, Sweden; 6 Department of Orthopaedics, Institute of Clinical Sciences, University of Gothenburg, Gothenburg, Sweden

**Keywords:** Cervical spondylotic myelopathy, Degenerative cervical myelopathy, Laminectomy, Cervical fusion, Reoperations, Randomized controlled trial

## Abstract

**Background:**

Cervical spondylotic myelopathy (CSM) is the predominant cause of spinal cord dysfunction in the elderly. The patients are often frail and susceptible to complications. Posterior surgical techniques involving non-fusion are complicated by postlaminectomy kyphosis and instrumented fusion techniques by distal junction kyphosis, pseudarthrosis, or implant failure. The optimal surgical approach is still a matter of controversy.

Since anterior and posterior fusion techniques have been compared without presenting any superiority, the objective of this study is to compare stand-alone laminectomy with laminectomy and fusion to determine which treatment has the lowest frequency of reoperations.

**Methods:**

This is a multicenter randomized, controlled, parallel-group non-inferiority trial. A total of 300 adult patients are allocated in a ratio of 1:1. The primary endpoint is reoperation for any reason at 5 years of follow-up. Sample size and power calculation were performed by estimating the reoperation rate after laminectomy to 3.5% and after laminectomy with fusion to 7.4% based on the data from the Swedish spine registry (Swespine) on patients with CSM. Secondary outcomes are the patient-derived Japanese Orthopaedic Association (P-mJOA) score, Neck Disability Index (NDI), European Quality of Life Five Dimensions (EQ-5D), Numeric Rating Scale (NRS) for neck and arm pain, Hospital Anxiety and Depression Scale (HADS), development of kyphosis measured as the cervical sagittal vertical axis (cSVA), and death. Clinical and radiological follow-up is performed at 3, 12, 24, and 60 months after surgery. The main inclusion criterium is 1–4 levels of CSM in the subaxial spine, C3–C7. The REDcap software will be used for safe data management. Data will be analyzed according to the modified intention to treat (mITT) population, defined as randomized patients who are still alive without having emigrated or left the study after 2 and 5 years.

**Discussion:**

This will be the first randomized controlled trial comparing two of the most common surgical treatments for CSM: the posterior muscle-preserving selective laminectomy and posterior laminectomy with instrumented fusion. The results of the myelopathy randomized controlled (MyRanC) study will provide surgical treatment recommendations for CSM. This may result in improvements in surgical treatment and clinical practice regarding CSM.

**Trial registration:**

ClinicalTrials.gov NCT04936074. Registered on 23 June 2021

**Supplementary Information:**

The online version contains supplementary material available at 10.1186/s13063-023-07123-4.

## Administrative information

Note: the numbers in curly brackets in this protocol refer to SPIRIT checklist item numbers. The order of the items has been modified to group similar items (see http://www.equator-network.org/reporting-guidelines/spirit-2013-statement-defining-standard-protocol-items-for-clinical-trials/).

**Table:** 

**Title {1}**	Comparison of posterior muscle-preserving selective laminectomy and laminectomy with fusion for treating cervical spondylotic myelopathy: study protocol for a randomized controlled trial
**Trial registration {2a and 2b}**	Clinical trials.gov identifier NCT04936074
**Protocol version {3}:**	220330; 2.1
**Funding {4}:**	This study is supported by the Uppsala, Stockholm and Gothenburg County Councils.
**Authors details {5a}**	1) Anna MacDowall, MD, PhD. Corresponding author. Address: Entrance 61, 6^th^ floor, Department of Surgical Sciences, Uppsala University, 75185 Uppsala, Sweden. Tel: +46730687087. E-mail address: anna.macdowall@surgsci.uu.se 2) Håkan Löfgren, MD, PhD. Neuro-Orthopedic Center, Jönköping, Jönköping County, and Department of Biomedical and Clinical Sciences, Linköping University, Linköping, Sweden, 3) Erik Edström, MD, PhD. Department of Neurosurgery, Karolinska University Hospital, Stockholm, Sweden and Department of Clinical Neuroscience, Karolinska Institutet, Stockholm, Sweden. Capio Spine Center Stockholm, Upplands-Väsby, Sweden. 4) Helena Brisby, MD, PhD. Department of Orthopaediscs, Institute of Clinical Sciences, University of Gothenburg, Gothenburg, Sweden. 5) Catharina Parai, MD, PhD. Department of Orthopaedics, Institute of Clinical Sciences, University of Gothenburg, Gothenburg, Sweden. 6) Adrian Elmi-Terander, MD, PhD. Capio Spine Center Stockholm, Upplands-Väsby, Sweden.
**Name and contact information for the trial sponsor {5b}**	No sponsor
**Role of sponsor {5c}**	No funder played any part in study design; collection, management, analysis, and interpretation of data; writing the report; and the decision to submit the report for publication.

## Introduction

### Background and rationale {6a}

Cervical spondylotic myelopathy (CSM) is characterized by weakness and paresthesia of the extremities, neck pain and stiffness, sphincter disturbance, and balance disorder. CSM is the most common cause of spinal cord dysfunction in the elderly [1] with an incidence of 41 per million in North America [2], peaking during the 7th decade of life. The male-to-female ratio is 2.7:1, and the most common level is C5–C6 [3].

CSM is typically the consequence of degenerative disc herniation, osteophyte formation, and hypertrophy of the ligamentum flavum that compress the spinal cord. Ossification of the posterior longitudinal ligament (OPLL), which is more prevalent in the Asian population, may also cause compression of the spinal cord. With non-operative treatments, i.e., medication and physiotherapy, 20–60% of the patients deteriorate neurologically and surgical treatment is therefore indicated [4].

The surgical treatment for CSM is decompression of the spinal cord. Decompression may be achieved with an anterior or posterior approach. Several algorithms have been proposed on whether to choose anterior discectomy and fusion, anterior corpectomy and fusion, posterior laminectomy with fusion, posterior laminoplasty, or posterior laminectomy alone [5, 6]. Anterior discectomy/corpectomy with fusion has been recommended in patients with a straight or kyphotic spine with compression of less than three levels [6]. A posterior approach has been recommended in patients with cervical lordosis and compression of more than three levels [7]. The World Federation of Neurological Societies (WFNS) Spine Committee modified these recommendations in 2019 towards a wider use of posterior approaches, e.g., in patients with posterior compression at 1 or 2 levels and patients with a flexible kyphosis [8]. It was recommended to address anterior compression with an anterior approach and posterior compression by a posterior approach.

A kyphosis leading to a cervical sagittal vertical axis (cSVA) of > 40 mm [9] (normal cSVA = 17–11 mm) [10] has been correlated to worse postoperative outcome in CSM patients, leading to the recommendation to correct kyphosis by an anterior approach [8]. In opposite to this, kyphosis correction has not shown to improve the clinical outcomes [11], indicating more remaining knowledge gaps concerning CSM patients with deformity.

Anterior and posterior decompression and fusion techniques have been compared with large sample sizes without presenting any difference in outcome [12, 13]. The question remains if fusion surgery is at all necessary as an adjunct to the decompression of the spinal cord and if CSM patients and, in that case, which CSM patients benefit from fusion surgery. After reports of postlaminectomy kyphosis in the 1970s and 1980s [14], prophylactic fusion has commonly been combined with the laminectomy procedure [12]. In a report from 1999, 34% of the patients developed kyphosis or swan neck deformity after laminectomy compared with 7% of patients surgically treated with laminoplasty, using a muscle-preserving technique [15].

A muscle-preserving technique that retains the facet integrity as well as the extensor musculature may also be used when performing posterior laminectomy. It has been observed to maintain sagittal balance after surgery without progression of kyphosis [16].

Posterior laminectomy as well as posterior laminectomy with fusion are both well-established surgical approaches. The most common surgical risks of both treatments are postoperative infection, dural tear, postoperative hematoma, neurologic deterioration, and C5 palsy. Specific risks of the muscle-preserving selective laminectomy are continuous degeneration of the affected level and progressive kyphotic deformity. Instrumented fusion may entail specific risks in terms of screw misplacement, implant failure, pseudarthrosis, increased surgical time, and blood loss [8]. Distal junction kyphosis (DJK) is a kyphotic angulation of at least 10° at the distal segment adjacent to a fused level and occurs in 24% of patients within a year after fusion surgery [17]. Adjacent segment pathology (ASP) is the progression of degeneration at the levels adjacent to a fused level and may also necessitate reoperation with laminectomy and extended fusion surgery [18].

In the lumbar spine, fusion surgery has been associated with increased operation times, blood loss, resource use, risk of complications, and 30-day mortality, when compared with laminectomy alone [19, 20]. A retrospective study in the cervical spine with a small sample size presented no differences in outcome between fusion or non-fusion groups after laminectomy, but increased operation time and blood loss in the fusion group [21].

There is yet to be investigated if the far more invasive fusion surgery is necessary to prevent the progression of deformity after decompression of the spinal cord or if the risk of DJK, pseudarthrosis, and implant failure after fusion surgery is more dangerous for these elderly and often frail patient group with CSM.

### Objectives {7}

The objective is to perform a non-inferiority study comparing posterior muscle-preserving selective laminectomy with laminectomy and fusion, to evaluate differences in outcome including reoperations, surgical complications, patient satisfaction, functional scores, and late degenerative changes including spondylolisthesis, kyphosis, and ASP.

We hypothesize that posterior muscle-preserving selective laminectomy is at least as successful in treating patients with CSM resulting in fewer reoperations, shorter hospital stay, and faster return to an active life, without reduced patient satisfaction, functional scores, or delayed kyphosis. Long-term follow-up radiographs and magnetic resonance imaging (MRI) are needed to assess differences in the subsequent degenerative changes including spondylolisthesis, kyphosis, and adjacent segment pathology (ASP) to compare the two strategies. Additionally, data from this study may be used to identify risk factors for the poor outcome to guide surgical decision-making.

### Trial design {8}

The MyRanc study is a multicenter randomized, controlled, parallel-group non-inferiority trial with 300 adult patients allocated in a ratio of 1:1 to two treatment groups: posterior muscle-preserving selective laminectomy (L-group) and laminectomy with instrumented fusion (LF-group).

## Methods: participants, interventions, and outcomes

### Study setting {9}

Participating centers are the University Hospitals of Uppsala (primary site), Stockholm and Gothenburg, and the County Hospital of RyHov in Jönköping. Each center has high volumes of CSM patients and complex spine surgery teams to provide the interventions.

### Eligibility criteria {10}

All patients diagnosed with CSM referred for a surgical consultation to the orthopedic or neurosurgery departments at the participating centers will be eligible for inclusion in the study. Patients will be enrolled if they meet the inclusion criteria (Table 1) and sign informed consent.

**Table 1:** Inclusion and exclusion criteria

Inclusion criteria	Age >18 years
	1–4 levels of cervical spondylotic myelopathy in the subaxial spine, C3–C7, without or with deformity not exceeding exclusion criteria, see below
	Eligible for both treatments
	Ability to understand and read the Swedish language
	Symptomatic myelopathy with at least one clinical sign of myelopathy
	No previous cervical spine surgery or thoracic/lumbar spinal stenosis surgery
	Psychosocially, mentally, and physically able to fully comply with this protocol, including adhering to scheduled visits, treatment plan, completing forms, and other study procedures
	Personally, signed and dated informed consent document prior to any study-related procedures, indicating that the patient has been informed of all pertinent aspects of the trial
Exclusion criteria	Local kyphosis; a modified K-line minimum interval distance (INT) of <4 mm [22]
	Spondylolisthesis >4 mm and simultaneous translation >2 mm on lateral flexion/extension radiographs [23]
	Soft disc herniations only (no signs of osteophyte formation and hypertrophy of the ligamentum flavum)
	Active infection
	Neoplasm
	Trauma
	Inflammatory disease (i.e., rheumatoid arthritis or ankylosing spondylitis or DISH)
	Systemic infectious disease including HIV
	Lumbar or thoracic spinal disease to the extent that surgical consideration is probable or anticipated within 6 months after the cervical surgical treatment (significant lumbar stenosis as defined by Schizas C or worse) [24]
	OPLL
	Parkinson’s disease
	Drug abuse, dementia, or other reasons to suspect poor adherence to follow-up
	Previous cervical spine surgery

### Who will take informed consent? {26a}

The consultant spine surgeon informs the patient who is eligible for inclusion of the study and hands out the written participant information. The research personnel follows up with the consent form a few days later. In this way, the patient may take some time to deliberate and subtle coercion will be minimized.

### Additional consent provisions for collection and use of participant data and biological specimens {26b}

No other consent provisions are applicable; no biological specimens are collected.

## Interventions

### Explanation for the choice of comparators {6b}

Considering the existence of muscle-preserving laminectomy techniques that can maintain cervical lordosis [16], there are reasons to explore the additional value of instrumented fusion in the cervical spine. Although both methods are widely used, they are yet to be compared in a randomized controlled study.

### Intervention description {11a}

#### General set up

Under general anesthesia, the patient is placed in the prone position and the head is fixed in a clamp. The appropriate levels are verified by fluoroscopy when needed. Local anesthetics are applied to the planned incision site.

#### Muscle-preserving selective laminectomy

A posterior midline incision is made, and the nuchal fascia is divided longitudinally. The spinous process is split using a high-speed drill/ultrasound knife and without disturbing the bilateral deep extensor muscles, the spinous process is divided at its base. Laminectomy is performed with a width not extending more than 2 mm outside the dural borders. The bilateral facet joints are not exposed. Finally, the split fragments of the spinous process are sutured together [16]. No collar or restrictions will be used [25].

#### Posterior decompression and fusion

A midline incision is made over the appropriate levels, defined as the extension of laminectomy without extending beyond C3–C7 [26]. Soft tissue dissection and retraction are performed to identify osseous landmarks. Special care is taken to spare muscle attachments on C2 and C7. Spinal instrumentation is performed with lateral mass or pedicle screws (C3–C7) combined with rod fixation. Laminectomy is performed with a width not extending more than 2 mm outside the dural borders. Facet joint injury should be avoided. Special care is taken to spare the C7 spinous process and the distal half of the C7 lamina [27]. The sagittal alignment is corrected before spinal fixation. No collar or restrictions will be used [25].

### Criteria for discontinuing or modifying allocated interventions {11b}

Both stand-alone laminectomy and laminectomy with fusion have been performed on a regular basis for decades. Still, if severe adverse events are noted, the attending spine surgeon will contact the investigator of the study. Analysis of severe adverse events will be conducted to evaluate if the method is harmful and if the trial needs to be discontinued.

### Strategies to improve adherence to interventions {11c}

The representative from the steering committee on each research site as well as the coordinator and controller will monitor that the participants get their allocated surgical treatment. After the surgery procedure, the postoperative treatments are the same for both groups, so no further compliance to interventions is needed except for monitoring the indications for reoperations which are defined in the “Outcomes {12}” section.

### Relevant concomitant care permitted or prohibited during the trial {11d}

Postoperative care, pain control, and physiotherapy will be identical in both groups. Stiff neck collars that affect the range of motion are prohibited.

### Provisions for post-trial care {30}

The participants get no provision for participating in the trial. General standard insurance in case of malpractice and harm is provided by the Swedish healthcare system as well as any further post-trial care, if required.

### Outcomes {12}

The primary endpoint is reoperation for any reason at 5 years of follow-up. Reoperation will be considered in case of:

- Postoperative hematoma or reperfusion injury with neurologic deterioration within hours/days after the primary surgery
- Change in sagittal alignment (kyphosis, DJK of more than 40 mm cSVA [9] and/or C2–C7 Cobb < −10°) with corresponding symptoms of camptocormia/increased pain/neurological deterioration
- ASP defined as degenerative changes on an adjacent level diagnosed with MRI and concomitant symptoms of myelopathy and/or radiculopathy
- Implant failure (clear radiolucency around >1 screw or rod breakage with increased neck pain and/or neurologic deterioration)
- Postoperative infection that requires revision surgery

Secondary outcome measures will be:

1. Change from baseline in myelopathy score on the patient-derived modified Japanese Orthopaedic Association scale (P-mJOA) at 2 years of follow-up
  - Change of at least 1 in mild myelopathy (>14 p), 2 in moderate myelopathy (12–14 p), and 3 in severe myelopathy (<12 p)
2. Change from baseline in participant’s disability score on the Neck Disability Index (NDI) at 2 years of follow-up
  Change of at least 17% [28] in the NDI (0–100%) compared with baseline (adjustable according to results from own minimal clinically important change results for NDI)
3. Change from baseline in myelopathy score on the Nurick scale at 2 years
4. Change from baseline in the patient Quality of Life Five Dimensions (EQ-5D index) at 2 years
5. Change from baseline in pain scores on the Numeric Rating Scale (NRS) at 2 years
  Pain relief, as defined by ≥2.5 points [29] improvement on a 10-graded numeric rating scale for arm/shoulder pain
6. Change from baseline in the number of participants with psychological impairment on the Hospital Anxiety and Depression Scale (HADS) in both groups at 2 years
  - The cutoff for depression is defined as >10 p [30]
7. Change in the number of satisfied participants on the satisfaction index in both groups at 2 years
8. Change from baseline in myelopathy on the 10-s grip and release test at 2 years
9. Change from baseline in myelopathy on the 10-s foot-tapping speed test at 2 years
10. Difference in the number of participants with treatment adverse events in both groups at 5 years
11. Change from baseline in sagittal alignment described as cSVA and C2–C7 Cobb angle in neutral radiographs at 5 years
12. Number of participants with a change in adjacent segment pathology (ASP) on MRIs from baseline to 5 years of follow-up
13. Number of participants with a change in compression of the spinal cord on the index level on MRIs from baseline to 5 years of follow-up
14. Change in direct costs between the groups at 1 year of follow-up
15. Change in indirect societal costs at 1 year of follow-up
16. Mortality at 5 years of follow-up

### Participant timeline {13}

The participant timeline is presented in Table 2.

**Table 2:** SPIRIT figure

Timepoint	Study period						
	Enrolment	Day of intervention	Post-allocation				
	−4–18 weeks	0	1 day	3 months	1 y	2y	5y
Enrolment							
Eligibility screen	X						
Informed consent	X						
Allocation	X						
Interventions							
Laminectomy or laminectomy with fusion		X					
Assessments							
Baseline variables	X						
Follow-up variables					X	X	X
X-ray	X				X	X	X
MRI	X			X			X
CT	X		X	X			X

### Sample size {14}

Based on data from the national Swedish spine registry on patients with CSM, reoperation was estimated to be 3.5% after stand-alone laminectomy and 7.4% after laminectomy and fusion. Five-year mortality was estimated to be 16.3% in the same population [31].

We further determined that excluding a 5% excess rate of reoperation in the laminectomy group vs laminectomy and fusion was a clinically relevant target for the study and therefore set the non-inferiority margin at 5 percentage points (pp).

With a planned sample size of 300 participants and with regard to mortality and an additional 5% loss due to dropout and emigration, we end up with 236 analyzable patients. This results in a power of 86% based on simulation using rerandomization. The reason for stopping at 300 patients was made on the calculation on how many patients it is reasonable for the four sites to include in 4–5 years. Ideally, we would have reached for 90% power but have agreed to compromise with a power over 85% if that means that we will finish the study in due time.

All statistical analyses were performed in R [32], version 4.0.5 (R Foundation for Statistical Computing, Vienna, Austria), and the R code used in the sample size calculation may be received upon request.

### Recruitment {15}

Patients with CSM are referred by a general practitioner to a consulting spine surgeon at a large center. Four large spine centers in Sweden are participating in the trial with patient enrolment to secure the reach of the target sample size. No specific strategy to promote the recruitment of patients is used.

## Assignment of interventions: allocation

### Sequence generation {16a}

Participants will be allocated to either stand-alone laminectomy or laminectomy and fusion through randomization with a 1:1 ratio using the REDcap software (Research Electronic Data Capture), after informed consent and agreement to be included in the study. After inserting the patient’s personal number into REDcap, the program reports the random allocation of the patient according to the pre-constructed randomization list. The randomization is stratified for center and participant sex, i.e., using separate lists for each center and sex. The allocation sequence utilizes balanced blocks of three different sizes occurring in a random sequence. The principal investigator and study collaborators are blinded to the sequence, the block sizes, and the block sequence.

### Concealment mechanism {16b}

Concealment mechanism is provided by the REDcap software that allows the user to perform randomization without the possibility of knowing the outcome beforehand. The statistician that created the randomization file that was imported to the REDcap randomization module is a separate statistician, not the same as the study statistician, and is otherwise not involved in the study.

### Implementation {16c}

One accountable spine surgeon at each center has individual logging to REDcap and the logon process is with a two-stage verification using logging and mail code. The accountable spine surgeon implements the allocation.

## Assignment of interventions: blinding

### Who will be blinded {17a}

Trial participants will not be blinded after assignment to interventions as they have online access to their medical records by a centrally managed system. Radiographic assessment is not possible to blind.

The outcome assessors and data analysts will be blinded by using a coding system for the participants and treatment groups.

### Procedure for unblinding if needed {17b}

No procedure for unblinding will be needed as all data analyses are performed after the trial is closed.

## Data collection and management

### Plans for assessment and collection of outcomes {18a}

Participant baseline data are collected manually before the intervention and entered into the REDcap software by the study nurse. Baseline data include routine demographics, smoking habits, work status, on sick leave, duration of neck pain, attitude towards returning to work, use of analgesics, fine motor skills, and patient-reported outcome measures (PROMs) including P-mJOA, NDI, EQ-5D, NRS separately for arm and neck pain, and HADS. The surgeon records data electronically including diagnosis, 10-s grip and release test, 10-s foot-tapping test, surgical treatment, surgical levels, neurological impairment, Nurick grading [33], instability, type of implant, operation time, blood loss, and any perioperative complications. Follow-up postal questionnaires and PROMs are sent to the participants after 1, 2, and 5 years, postoperatively, according to Swespine routines [34, 35].

#### Primary outcome

Assessment regarding the primary endpoint will be made at the 1, 2, and 5 years of follow-up (study protocol initiated) or at any timepoint when increased pain, deformity, or neurological deterioration is detected (patient initiated).

#### Secondary outcomes

The P-mJOA (continuous variable) is a self-administered questionnaire with four domains, measuring motor (upper and lower extremities), sensor (upper extremities), and sphincter dysfunction in participants with CSM. The first domain is scored from 0 to 5, the second domain from 0 to 7, and third and four domains from 0 to 3. The maximum score is 18 points, indicating no deficits and the minimum score is 0, total tetraplegia. The minimum clinically important difference (MCID) in mJOA has been reported to be 2 overall with a threshold of 1 in mild myelopathy (mJOA >14), 2 in moderate myelopathy (mJOA 12–14), and 3 in severe myelopathy (mJOA <12) [36]. Consistency between P-mJOA and mJOA is demonstrated with identical mean scores and a SD of 1.5 together with a strong agreement of 0.83 with the use of the intraclass correlation coefficient and the Spearman correlation [37].

The NDI [28] (continuous variable) ranges from 0 to 100% with higher scores indicating severe disability. The MCID is 15–17% [28, 29, 38].

The Nurick scale (categorical variable) has six grades (0–5) based on the “difficulty in walking.”

EQ-5D index (continuous variable) ranges from −0.5 to 1 with higher scores reflecting a better quality of life [34]. The MCID is 0.24 [28].

EQ VAS (continuous variable) ranges from 0 to 100 with higher scores indicating better health.

NRS for neck and arm pain [29] (continuous variable) ranges from 0 to 10, with higher scores indicating more severe pain. The MCID is 2.5 for NRS of the neck and arm [29].

HADS (continuous variable) ranges from 0 to 42 with higher scores indicating more anxiety/depression with a cutoff for depression of >10 p [30].

The satisfaction index (categorical variable) evaluates the participants’ experienced overall result after surgery. The participant is asked “How is your attitude regarding the treatment result.” The alternative answers are “satisfied,” “uncertain,” and “dissatisfied.”

The 10-s grip and release test (continuous variable) is performed with the forearm in pronation and the wrist in mild extension. The participant is asked to grip and release with their fingers as rapidly as possible and the number of completed cycles of movement within 10 s is counted. Both hands are tested separately. The test has been correlated to the JOA score and differences between sex and age groups in the normal population have been set [39].

The 10-s foot-tapping speed test (continuous variable) is performed with the participant sitting on a chair adjusted so that the hip and knee are flexed at 90° and the bilateral soles have contact with the floor. The participant is asked to move their toes up and down, tapping the floor as quickly as possible for 10 s with their heels firmly planted on the floor. The number of completed cycles of movement within 10 s is counted and both feet are tested separately. The test has been correlated to the JOA score, Nurick grade, 10-s grip and release test, and 30-m walking test [40].

All adverse events in both groups will be recorded. The adverse events will be graded according to severity (categorical variable): grade 1, any non-life-threatening complication treated without invasive procedures; grade 2, complications requiring invasive management such as surgical, endoscopic, and endovascular procedures; grade 3, life-threatening adverse events requiring treatment in an intensive care unit; and grade 4, death as a result of complications [41].

The C2–C7 Cobb angle, C2–C7 cSVA, C7 slope, and T1 slope (continuous variables) are all measured on images taken preoperatively and at 1, 2, and 5 years of follow-up [10, 42]. The development of ASP will be monitored. Participants who develop a progressive kyphosis/DJK of more than 40 mm cSVA [9] and/or C2–C7 Cobb < −10° and neurological deterioration/ASP/restenosis will undergo additional magnetic resonance imaging (MRI) and may be subject to supplementary posterior fusion/revision.

Direct costs include hospital stay related to the surgical procedure, implant-related costs, and pain medication usage. Indirect costs include pain medication usage after discharge, societal costs for absence from work, and worker compensation.

#### Imaging

Standing radiographs of the cervical spine in neutral, flexion, and extension will be performed preoperatively and at 1, 2, and 5 years of follow-up. When radiographs of the neutral position are executed, the participant will be in a comfortable standing position with the head facing forward for a horizontal gaze. The radiographs will be used to investigate the sagittal alignment by measuring the C2–C7 Cobb angle, C2–C7 cSVA, C7 slope, and T1 slope [16].

MRI of the cervical spine with T1- and T2-weighted images in sagittal and T2-weighted in axial planes will be performed preoperatively, after 3 months and at 5 years of follow-up. Hereby the compression of the spinal cord can be assessed as well as the performed decompression and the degenerative development over time.

Computed tomography (CT) will be performed pre- and postoperatively and at 3 months and 5 years to control the screw fixation, bony anatomy, and fusion (Table 2).

### Plans to promote participant retention and complete follow-up {18b}

Participants that do not reply on the first set of PROMs will get two reminders by mail. If still no response, they will be approached once by telephone. If still no response, information about missing participants that have not left the study will be retrieved from the medical records, radiographs, and the Swedish patient registry.

### Data management {19}

The REDcap software, with a two-stage verification login, will also be used for safe data management (project-redcap.org; redcap.vanderbilt.edu). The data will be pseudonymized and all participants will have a specific code. Access to decrypted data and safekeeping of the decryption key will be limited to the principal investigator and the study nurse. The principal investigator is responsible for communicating important protocol modifications and is together with the research nurse responsible for encrypting participant data and protecting the decryption key.

### Confidentiality {27}

All data managements and analysis are done using unidentified data. The participating spine surgeons will only have access to data generated on their own site and cannot see the other recruiting centers.

### Plans for collection, laboratory evaluation, and storage of biological specimens for genetic or molecular analysis in this trial/future use {33}

No laboratory test or biological materials are collected during this trial.

## Statistical methods

### Statistical methods for primary and secondary outcomes {20a}

All endpoints will be analyzed in the modified intention to treat (mITT) population, defined as randomized patients who are still alive without having emigrated or left the study after 2 and 5 years.

#### Primary outcome

To test for non-inferiority, a two-sided 95% confidence interval (CI) for the difference in failure (reoperation) rates between the two groups will be computed. To account for the sparsity of events, the CI will be computed using rerandomization techniques [43], blocked on sex, since the randomization was stratified on sex. More precisely, a null distribution for the test statistic, i.e., the difference in failure rates, under the null hypothesis that the non-inferiority margin is correct will be generated via permutations. The one-sided *p*-value is the proportion of simulated test statistics that are larger than the observed one. To generate the null distribution, the non-inferiority margin of 0.05 will first be subtracted from the observed 0/1 failure variables in the non-fusion group. These modified outcomes will then be permuted a large number of times, recording the difference in means between the groups for each permutation. Non-inferiority will be claimed if the upper limit of the CI is less than 5 pp. If non-inferiority is demonstrated, superiority will also be tested using the same CI, although the study is likely underpowered to detect this.

#### Secondary outcomes

The secondary outcomes will be analyzed using ordinal regression models, adjusted for sex. In addition, each secondary endpoint will be dichotomized and analyzed using logistic regression. The dichotomization will be done by comparing baseline and follow-up data, either based on MCID when applicable or else by defining success as an improvement from baseline. All secondary endpoints will be analyzed at 1, 2, and 5 years of follow-up, but not until the study is closed.

### Interim analyses {21b}

An interim analysis regarding adverse events and harms (see the section “Adverse events reporting and harms {22}” for definitions and grading) will be performed by an independent observer when 100 participants are included. The results will be reported to the principal investigator and the accountable researchers at each center and the group will make the decision to terminate the trial if the difference in adverse events and harms between the groups is statistically significant.

### Methods for additional analyses (e.g., subgroup analyses) {20b}

No primary subgroup analyses are planned.

### Methods in analysis to handle protocol non-adherence and any statistical methods to handle missing data {20c}

Dropouts may be one out of two entities: (1) the participant actively leaves the study or (2) the participant has died or does not show up on follow-ups for unclear reasons. In case 1, the participant will not be part of the study anymore and data will not be retrieved from other information sources. In case 2, information about living participants will be retrieved from the medical records, radiographs, and the Swedish patient registry. In case 2, missing PROMs will be imputed using multiple imputation [44]. Reoperations, adverse events, and radiographs will not be imputed.

### Plans to give access to the protocol, participant-level data, and statistical code {31c}

A deidentified dataset and the R code used for the statistics will be delivered upon request after the completion and publication of the 5-year follow-up.

## Oversight and monitoring

### Composition of the coordinating center and trial steering committee {5d}

The coordinating center is Uppsala University Hospital.

The steering committee is Anna MacDowall, study director and overall supervision of the study; Adrian Elmi-Terander and Erik Edström, responsible for the study site in Stockholm; Håkan Löfgren, responsible for the study site in Jönköping; and Helena Brisby and Catharina Parai, responsible for the study site in Gothenburg. The steering committee will have meetings once every 6 months.

The main coordinator and controller is Catarina Strömstedt, responsible for data managing in REDcap.

The independent observer is Prof. Nils Hailer, MD, PhD, responsible for interim analysis and auditing trial conduct.

The study sites have each a coordinator that is in direct contact with the main coordinator and controller.

The study statistician is Lars Lindhagen at Uppsala Clinical Research Center.

### Composition of the data monitoring committee, its role, and reporting structure {21a}

Data monitoring is performed on a weekly basis by the main coordinator and controller with the support of the local coordinator to ensure that all events, PROMs, and radiology are performed according to the protocol.

The data monitoring committee comprises the study director and the main coordinator and controller. The monitoring committee is independent from sponsors and competing interests.

### Adverse event reporting and harms {22}

As reoperations due to complications and adverse events are the primary outcome, all adverse events in both groups will be recorded. The adverse events will be graded according to severity (categorical variable): grade 1, any non-life-threatening complication treated without invasive procedures; grade 2, complications requiring invasive management such as surgical, endoscopic, and endovascular procedures; grade 3, life-threatening adverse events requiring treatment in an intensive care unit; and grade 4, death as a result of complications [41].

The adverse events are reported by the senior spine surgeon to the steering committee.

### Frequency and plans for auditing trial conduct {23}

Auditing trial conduct will be performed by the independent observer, and an interim analysis will be made when 100 participants are included.

### Plans for communication of important protocol amendments to relevant parties (e.g., trial participants, ethical committees) {25}

Any change in the protocol from the primary protocol that is approved by the Swedish Ethical Review Authority must be resubmitted for ethical approval and reported to relevant parties such as trial participants and trial registries.

### Dissemination plans {31a}

The trial result will be submitted to peer review journals and presented at international meetings.

## Discussion

Cervical spondylotic myelopathy is the most common cause of spinal cord dysfunction in the elderly worldwide [1]. In the twentieth century, laminectomy was regarded as the standard posterior approach for the treatment of CSM but fell into disfavor due to reports of postlaminectomy kyphosis in the 1970s and 1980s [25]. Since then instrumentation techniques for spinal fixation and fusion have developed and spine research has become an industry-driven field [45]. Posterior laminectomy is now commonly combined with prophylactic fusion even in patients with a lordotic cervical spine [12]. Laminoplasty was developed as a motion-preserving technique for posterior decompression in patients with CSM [46]. The technique retains the facet integrity as well as preserves the extensor muscles and has equal or better results compared with posterior laminectomy and fusion [47]. These comparisons indicate that it is not the laminectomy itself that may lead to postoperative kyphosis but the damage to other important structures like muscles and facet joints. With a technique to spare these structures, instrumentation may be redundant. The research group of Professor Shiraishi has described the posterior muscle-preserving selective laminectomy technique and observed that the patients maintain sagittal balance after surgery without any progression of kyphosis [16]. However, the technique has not been subjected to any well-designed clinical trials.

The term “instability” has been defined as “loss of the spine’s ability to maintain its patterns of displacement under physiologic loads so there is no initial or additional neurologic deficit, no major deformity, and no incapacitating pain” [48]. The cutoff values most commonly used to realize this description into practice are a translational movement of > 3.5 mm, an angulation of > 11°, and < 50% facet joint covering on flexion-extension radiographs [23]. Thus, when Panjabi and White wrote these cutoff values in the 1970s, their cadaver studies described trauma to the cervical spine, not degenerative changes. Specific cutoff values for “instability” in the nontraumatic degenerated cervical spine are yet to be defined.

The anterior versus posterior decompression and fusion approaches have been compared without showing any differences in postoperative results [12, 13]. The anterior approach is often preferred to restore sagittal alignment [25]. As in the lumbar spine, the loss of lordosis may be a compensatory mechanism to create more space in the narrowed spinal canal. After decompression, the conditions are reversed and the lordosis may be restored as long as the muscles and the facets are preserved [16]. Thus, adding fusion to the decompression surgery would be unnecessary. Nonetheless, a modified K-line INT < 4 mm has been observed to be disadvantageous for a posterior approach and thus needs to be regarded as a guideline cutoff value [22].

Until now, there is no consensus on whether to fuse or not when laminectomy is performed, and the choice of surgical method is dependent on the surgeon’s preference. This will be the first randomized controlled trial comparing two common surgical treatments for CSM: the posterior muscle-preserving selective laminectomy and posterior laminectomy with instrumented fusion.

## Trial status

The trial started in February 2022 with four sites in Sweden, the Department of Surgical Sciences, the Academic University Hospital in Uppsala; the Department of Neurosurgery, Karolinska University Hospital in Stockholm; the Neuro-Orthopaedic Center, Ryhov Hospital in Jönköping; and the Department of Orthopaedics, Sahlgrenska University Hospital in Gothenburg. More sites may be recruited during the enrollment phase. The expected enrollment is 5 years, and the conclusion of the study is estimated at 10 years.

## Supplementary Information

**Additional file 1.** The patient informed consent form.

## Data Availability

Access to decrypted data and safekeeping of the decryption key will be limited to the principal investigator and the study nurse. Participating spine surgeons only have access to their own site data and cannot see the data of the other recruiting centers. The use of encrypted data sets and the RedCap software management program will protect confidentiality. Anonymized data can be provided upon request.

## Abbreviations

ASP: Adjacent segment pathology
BMI: Body mass index
CI: Confidence interval
CSM: Cervical spondylotic myelopathy
cSVA: Cervical sagittal vertical axis
CT: Computed tomography
DISH: Diffuse idiopathic skeletal hyperostosis
EQ-5D: European Quality of Life Five Dimensions
HADS: Hospital Anxiety and Depression Scale
HIV: Human immunodeficiency virus
ITT: Intention to treat
K-line INT: Kyphosis-line minimum interval distance
MCID: Minimal clinically important difference
MRI: Magnetic resonance imaging
NDI: Neck Disability Index
NRS: Numeric Rating Scale
OPLL: Ossification of the longitudinal ligament
P-mJOA: Patient-derived modified Japanese Orthopaedic Association scale
PP: Per protocol
PROM: Patient-reported outcome measure
REDcap: Research Electronic Data Capture
SD: Standard deviation
Swespine: Swedish spine registry
