# Supplementary material for: Comparison of posterior muscle-preserving selective laminectomy and laminectomy with fusion for treating cervical spondylotic myelopathy: study protocol for a randomized controlled trial
Source: Trials. 2023 Feb 11;24:106. doi: 10.1186/s13063-023-07123-4 (PMC9921403; doi:10.1186/s13063-023-07123-4)
Supplement: Supplementary file 1 [file 13063_2023_7123_MOESM1_ESM.docx]

INFORMED CONSENT FORM

The Swedish myelopathy study

Myelopathy **Ran**domized **C**ontrolled trial – MyRanC

Fusion or no fusion after cervical decompression of the spinal cord stenosis

Consent to participation in a research project

☐ I have received information about the research project "The Swedish myelopathy study".

☐ I am aware that my participation in the study is voluntary. I know that if I decline participating in the study it will not affect the health care provided to me and that I might discontinue my participation whenever I want without having to explain why.

☐ By participating in the study, I approve of the setup of a computer register containing my personal data and some clinical data (like the results from radiographs and diagnoses). Except from the researchers participating in the study no other persons or organizations will have access to this data.

City ______________________ Date ______________________

Signature ______________________

Name ______________________

I have verbally explained the study and its purpose to the participant.
The participant has had the opportunity to ask questions and to get answers.
After signing the participant gets one copy of the informed consent form.

Physician/Study nurse signature ______________________ Date

Physician/Study nurse name ______________________
